# Towards Minimizing the LiDAR Sim-to-Real Domain Shift: Object-Level Local Domain Adaptation for 3D Point Clouds of Autonomous Vehicles

**DOI:** 10.3390/s23249913

**Published:** 2023-12-18

**Authors:** Sebastian Huch, Markus Lienkamp

**Affiliations:** Institute of Automotive Technology, Munich Institute of Robotics and Machine Intelligence (MIRMI), Technical University of Munich, 85748 Garching, Germany; lienkamp@tum.de

**Keywords:** autonomous vehicles, deep learning, domain adaptation, LIDAR, object detection, point cloud, synthetic data

## Abstract

Perception algorithms for autonomous vehicles demand large, labeled datasets. Real-world data acquisition and annotation costs are high, making synthetic data from simulation a cost-effective option. However, training on one source domain and testing on a target domain can cause a domain shift attributed to local structure differences, resulting in a decrease in the model’s performance. We propose a novel domain adaptation approach to address this challenge and to minimize the domain shift between simulated and real-world LiDAR data. Our approach adapts 3D point clouds on the object level by learning the local characteristics of the target domain. A key feature involves downsampling to ensure domain invariance of the input data. The network comprises a state-of-the-art point completion network combined with a discriminator to guide training in an adversarial manner. We quantify the reduction in domain shift by training object detectors with the source, target, and adapted datasets. Our method successfully reduces the sim-to-real domain shift in a distribution-aligned dataset by almost 50%, from 8.63% to 4.36% 3D average precision. It is trained exclusively using target data, making it scalable and applicable to adapt point clouds from any source domain.

## 1. Introduction

Autonomous vehicles (AVs) are changing the automotive industry, promising improved road safety and a reduction in emissions [1]. Autonomous shuttles are already operating in restricted areas on public roads, even without safety drivers, but this approach to AVs does not scale well. AVs are equipped with sensor arrays, including cameras, LiDAR sensors, and RaDAR sensors, and are based on data-centric neural networks to perform tasks such as object detection. While training of these object detection networks is usually performed when supervised, the choice and characteristics of the datasets play a crucial role in the network’s real-world performance.

The performance of perception algorithms in autonomous vehicles is related to the quality and quantity of the dataset used for training [2,3,4,5]. This performance limitation is known as the data hunger effect [6]. Specifically, the algorithms’ ability to accurately detect and interpret objects in diverse real-world scenarios is based on the datasets’ representation of these conditions. Factors such as differences in the environmental conditions, types of objects, and sensor inaccuracies in the datasets significantly influence the algorithms’ resilience and ability to adapt to these conditions in the real world. This interdependence highlights the need to select diverse and well-curated datasets to develop reliable perception algorithms for AVs. Thus, the selection of a dataset is a critical factor in the overall success of autonomous vehicle perception.

Pairs of images or point clouds and their corresponding labels are required to train an object detection network. Furthermore, to train these networks effectively, it is necessary to have a large amount of labeled data similar to the conditions that will be encountered when the network is used in the real world. When networks are trained in one domain and then applied to another, for example, when using different datasets from the real world, such as KITTI [7], Waymo [8] or nuScenes [9], a real-to-real domain shift can be observed [10,11,12]. To be successful in inference, there must be a high degree of domain similarity between the source dataset used for training and the target data seen during inference. In conclusion, the scalability of AVs is highly dependent on the datasets needed for the perception algorithms.

Gathering and labeling real-world data is a time-consuming and costly undertaking, as it is a manual process and requires additional efforts for each new sensor configuration. An alternative to real-world data is to employ synthetic data created in 3D simulation environments, such as CARLA [13]. This approach is highly scalable, as the data are automatically labeled in the simulation and further safety-critical scenarios can be recorded in the simulation.

However, the divergence between simulation data and real-world data presents unique challenges. Simulation environments such as CARLA, while highly controlled and reproducible, often lack the complex and unpredictable nature of the real world. Key differences include the representation of environmental conditions, such as varying light and weather conditions, and the dynamic behavior of other road users and pedestrians. Data from simulations typically feature more idealized and consistent conditions, whereas real-world data encompass a wide range of variability, i.e., unexpected sensor noise and the often erratic behavior of humans in the real world. Additionally, the physical properties of objects, such as textures and reflectivity, are often simplified in simulations, leading to a gap in the fidelity of sensor data, particularly for LiDAR and camera sensors. This disparity poses significant challenges in training and validating perception systems, as algorithms developed and tested in simulations may not translate effectively to the complexities and unpredictability encountered in real-world environments.

Similarly to the real-to-real domain shift, a sim-to-real domain shift can also be observed. This occurs when object detection networks are trained with synthetic data and deployed in the real world. The source of the sim-to-real domain shift is a combination of various factors. These include the difference in data distribution, diversity, and lack of realism of the virtual sensor model [14], as it is not an exact replication of the physical sensor and its associated characteristics.

Several studies have been conducted to evaluate or quantify the sim-to-real domain shift, using camera [15,16] or LiDAR [17,18] object detection networks for quantification. The authors train multiple networks on a source dataset and evaluate the trained networks on a target dataset to quantify the domain shift. A metric, such as the mean average precision (mAP), is used to calculate the object detection performance, which can be compared to the object detection performance of a network trained and evaluated on the target dataset. For instance, the study [17] shows that the LiDAR sim-to-real domain shift can be as large as 50%, with real-world trained models achieving more than 70% mAP, while models trained with simulated data consistently reach less than 20% mAP on the real-world test dataset. These studies underscore the significance of the sim-to-real domain shift and its implications for safety-critical applications, such as autonomous vehicles. In other words, despite the advantages of using simulation data, these synthetically generated data cannot be used for real-world applications, and expensive real-world data still need to be collected and annotated.

As this is not scalable for a large number of perception algorithms, the research field of domain adaptation has emerged. Domain adaptation is a form of transfer learning that aims to minimize the domain shift between datasets. A variety of strategies for domain adaptation exist to reduce the domain shift, which will be reviewed in more detail in the following chapter.

Our work presents a new domain adaptation approach that adapts point clouds on the object level. The focus of our algorithm is to minimize the LiDAR sim-to-real domain shift, which arises specifically from differences in sensor noise between the simulation and the real world and is, therefore, limited to local regions. The goal of our algorithm is not to minimize the domain shift caused by differences in scenarios or the distributions of vehicle shapes and sizes. In detail, our main contributions are as follows:

- We propose a domain adaptation approach that adapts a LiDAR point cloud on the object level, open-source available at https://github.com/TUMFTM/LOL_DA (accessed on 21 November 2023).
- Our domain adaptation network is trained unsupervised and our modular architecture is based on a point completion network, making it easily interchangeable with other point completion networks.
- A distinctive aspect of our approach is the focus on local adaptation by employing downsampling to generate domain-invariant representations of the object point clouds and using a discriminator working with point cloud patches.

## 2. Related Work

In this chapter, we discuss the research fields of domain shift and domain adaptation, with a particular emphasis on methods for adapting LiDAR point clouds.

### 2.1. Domain Shift

To assess the efficacy of any domain adaptation technique, it is necessary not only to qualitatively examine the adapted samples, but also to quantify the domain shift using the generated samples in a perception task, such as object detection or segmentation [19]. In order to measure the domain shift, perception networks are trained on both the source and target domains and then evaluated on the target domain. This can be performed for object detection [17] or semantic segmentation [11,12]. Training with the adapted dataset and evaluating on the target domain, one can determine the effectiveness of the domain adaptation method [20,21], which, in turn, determines the degree of reduction in the domain shift. To quantify the domain shift specifically for the sim-to-real setting, a method has been introduced that focuses on the local differences between the simulation and the real world by using a scenario-aligned dataset, providing the same scenes in the real world and the simulation [18]. In this study, we will use this dataset to measure the effectiveness of the presented domain adaptation method. An additional way to evaluate the effectiveness of domain adaptation algorithms is to compare the latent spaces produced by a variational autoencoder (VAE) [22]. In [18], we also compare latent spaces, but instead of using a distinct VAE network, we employ our pretrained perception networks to generate the latent feature vectors. These high-dimensional latent feature vectors can be visualized using methods such as t-distributed stochastic neighbor embedding (t-SNE) [23].

### 2.2. Domain Adaptation

In general, domain adaptation is a form of transductive transfer learning [24]. This field of research is characterized by the fact that the source domain labels are available, whereas the target domain labels are not. The domain adaptation process only alters the data before passing them to the perception task, such as an object detection network, and does not modify the perception task itself. Since labeled target data are unavailable, it is also called unsupervised domain adaptation [25]. Domain adaptation can be further categorized into domain-invariant feature learning, normalization statistics, and domain mapping. This categorization originates in the image-based domain adaptation as defined in [19]. In addition to these categories, [25] further introduces the category domain-invariant data representation, another data-driven approach specifically for LiDAR-based domain adaptation. These four approaches to domain adaptation will be reviewed in the following.

**Domain-invariant feature learning** techniques attempt to make the features produced by a feature extractor independent of the domain, meaning that the features of the source and target domains should have the same distribution. To achieve this, there are two approaches: One is to reduce the divergence of features by employing batch statistics [26] or a cross-model loss from 2D images and 3D point clouds [27]. The other approach is to align the features using a discriminator [28,29].

**Normalization statistics** techniques employ specific forms of batch normalization layers, such as adaptive batch normalization (AdaBN). The idea is that batch norm statistics can acquire domain knowledge and can be transferred from the source to the target domain. However, ref. [30] highlights that simply relying on AdaBN does not result in satisfactory performance for cross-sensor domain adaptation.

Data preprocessing algorithms that create a domain-invariant representation of the source and target data are known as **domain-invariant data representation** methods. This representation is then used to train and evaluate a perception network. A two-dimensional projection can be used to convert the source and target data into a domain-independent representation [31,32,33]. Alternatively, a voxelization of the point cloud can be performed before it is fed into the perception network [34,35].

**Domain mapping** techniques can transform images or point clouds from the source domain to the target domain, which can then be used to train a perception network. The aim of these methods is to modify the source distribution to the target distribution without altering the source semantics, meaning that the source labels remain the same and can be used to train the perception network with pseudo-target samples. Research in this area is scarce and can be divided into two approaches, both of which are based on unaltered generative adversarial network (GAN) techniques created for image-to-image conversion. The first category is to create 2D birds-eye-view image projections from 3D point clouds before passing these into the domain mapping algorithm, such as CycleGAN [36]. For example, refs. [37,38,39] use this approach to adapt synthetic point clouds to the real world. Since the output of this method is a 2D top-view projection, just like the input, a 3D point cloud cannot be recovered. The authors use YOLOv3 [40] for object detection to evaluate their methods using adapted 2D projections. Instead of using 2D projected data, the second approach of domain mapping is to generate front-view images as in [41,42]. Front-view images are a lossless projection of 3D point clouds. Therefore, it is possible to obtain 3D point clouds after translating the domains. Refs. [42,43] investigated the dropout of points from real-world point clouds and applied the same technique to synthetic point clouds. Rather than relying on image-based adversarial domain mapping, ref. [44] proposed aligning the data and class distributions of the source and target domains by means of augmentation techniques and minimizing the Kullback–Leibler divergence between the object classes. Ref. [45] employed a non-adversarial domain mapping technique for real-to-real sensor-to-sensor transfer, with a focus on semantic segmentation. The authors accumulated multiple annotated scans using 3D LiDAR SLAM to create a point cloud map, and then generated semi-synthetic scans from the accumulated map with different sensor parameters as the source sensor. There are methods to map the domains of 3D point clouds beyond the field of autonomous driving, as demonstrated in [46]. This paper introduces an unsupervised 3D domain adaptation technique for objects that aligns the source and target distributions both locally and globally. However, ref. [25] highlighted the scarcity of generative domain mapping methods that adapt 3D point clouds instead of 2D images. For this reason, we aim to close this research gap by proposing a novel domain mapping method. Instead of converting the 3D point clouds into an irreversible 2D bird’s-eye view or front-view representation as in [37,38,39,41,42], our method directly adapts point clouds in their 3D representation, leveraging the local relationships between neighboring points. Moreover, instead of adapting the entire scene point clouds and potentially altering the scene semantics as in previous methods, our method adapts point clouds on an object level and ensures the preservation of scene semantics.

## 3. Method

### 3.1. Problem Formulation and Notation

A point cloud is an unordered set of points $X_{i}=\left\{p_{j}{|}_{j=1}^{N_{j}}\right\}$ with $N_{j}$ points in each set, where $p_{j}=\left\{x_{j},y_{j},z_{j}\right\}\in R^{3}$ denotes the 3D coordinates of each point $p_{j}$ of the *i*-th point cloud. For each point cloud $X_{i}$, there is a set of annotations $Y_{i}=\left\{y_{k}{|}_{k=1}^{N_{k}}\right\}$, where $y_{k}={\left\{c_{x},c_{y},c_{z},l,w,h,\theta_{z}\right\}}_{k}$ denotes the center, dimension, and rotation around the *z*-axis of a 3D bounding box [12]. We focus our efforts on adapting at the object level, so we define the points within each bounding box $y_{k}$ of a scene point cloud $X_{i}$ as $O_{i,k}$, which denotes the *k*-th object point cloud of the *i*-th scene point cloud.

In unsupervised domain adaptation, the source domain $S=\{\left(X_{i}^{S},Y_{i}^{S}\right){|}_{i=1}^{N_{S}}\}$ is composed of $N_{S}$ point clouds $X_{i}^{S}$, each of which is annotated with the corresponding labels $Y_{i}^{S}$. The target domain $T=\{X_{i}^{T}{|}_{i=1}^{N_{T}}\}$ only contains $N_{T}$ point clouds $X_{i}^{T}$ and no annotations are provided. The objective of unsupervised domain adaptation is to learn a mapping function Ψ between the point clouds of the source domain $X_{i}^{S}$ and the target domain $X_{i}^{T}$.

### 3.2. General Outline

The purpose of our research is to train 3D object detection networks using adapted data from the source domain and to test these trained networks in the target domain. Therefore, the domain adaptation to reduce the domain shift between the source and target data is performed on the object level, as objects are the most relevant features for an object detection algorithm. To achieve this, only the objects $O_{i,k}$ labeled in the source dataset are adapted, not the entire scene point clouds $X_{i}^{S}$.

This focus on object-level adaptation is motivated by both practical and scientific considerations. The point clouds of entire scenes are usually too large to be processed efficiently on GPUs and exhibit greater sparsity than object point clouds. Additionally, our chosen network, designed primarily for point cloud completion, is optimized to process individual objects rather than entire scenes. Adapting entire scenes would risk losing crucial global structure within the objects, especially when FPS downsampling is taken into account. In comparison, object-level adaptation provides a more consistent density and better preservation of the global structure, which is in line with the abilities of our point completion network, and, thus, guarantees more accurate and meaningful domain adaptation.

A key aspect of our training methodology involves using the ground truth of the target scene. This approach, in contrast to traditional domain adaptation methods that tackle label scarcity in the target domain, enables our model to independently supervise the adaptation of source data. By taking this approach, our training is not dependent on the source domain, allowing the model to adapt any source domain without the requirement of retraining for each new domain. For instance, various datasets from different simulations can be adapted to the real-world style after a single training using a limited set of labeled target data. This approach can be advantageous as it boosts performance by utilizing large, easily generated simulated datasets; a limited number of labeled target data can enable the adaptation of a much larger set of source data.

Following the training phase, our domain adaptation approach demonstrates its scale capability, allowing the adaptation of an unlimited number of source point clouds from any source domain. This feature is particularly advantageous for sim-to-real domain adaptation scenarios, where simulation engines typically provide automatic annotation of point clouds.

**Figure 1:**
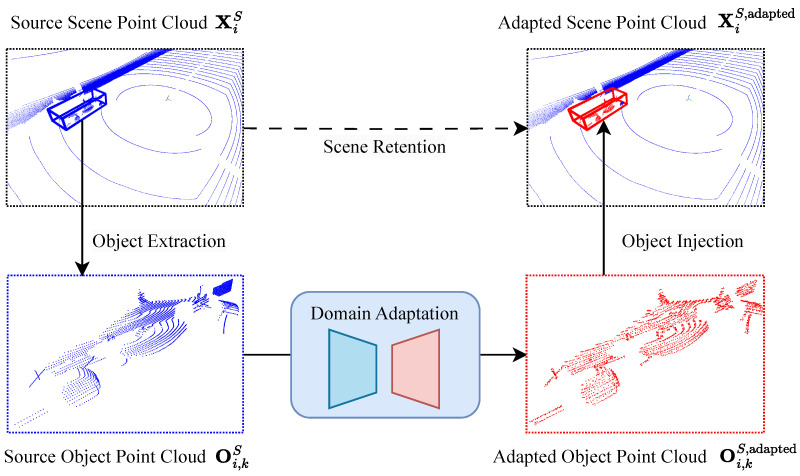
We present an object-based point cloud domain adaptation method. We start by extracting object point clouds $O_{i,k}^{S}$ from a source scene point cloud $X_{i}^{S}$ (here: simulated data, blue). Our trained domain adaptation network then adapts these object point clouds to create target-style object point clouds (here: real-world, red) $O_{i,k}^{S,\mathrm{adapted}}$, which are placed back in their original positions in the source scene point cloud. The final output $X_{i}^{S,\mathrm{adapted}}$ is a combination of the original source scene point cloud and the adapted object point cloud.

The illustration in Figure 1 outlines our general process, which is explained in the following. We start by obtaining the relevant objects $O_{i,k}$ from the source S and target T datasets, respectively. This is performed by extracting the objects from all scene point clouds $X_{i}$. Examples of these objects can include cars, pedestrians, and cyclists, which are defined by the object detection task. Using the extracted objects $O_{i,k}^{T}$ from the target domain, we train a point completion network to reconstruct these objects. The input to the network is a downsampled representation of the object point clouds, making them domain-invariant. In this way, our network learns to generate objects and applies the desired target characteristics locally while preserving the global structure of the input object. This is important because we do not want to alter the global structure of the objects, as they are reinserted into their original position in the scene point clouds during domain adaptation inference.

Once the network has been trained and, hence, has learned to reconstruct point clouds with target domain characteristics, we use source object point clouds $O_{i,k}^{S}$ as the input for the network during inference instead of the target object point clouds $O_{i,k}^{T}$. The source object point clouds are also downsampled to remove the local structure before being fed to the network, making them domain-invariant and, thus, allowing the network to generate target-style object point clouds while preserving the global structure of the source object point cloud. Finally, the adapted source object point clouds $O_{i,k}^{S,\mathrm{adapted}}$ are reinserted into their original positions in the source scene point cloud $X_{i}^{S}$. The adapted scene point clouds $X_{i}^{S,\mathrm{adapted}}$ are used to train object detection networks, with the hypothesis that this results in a reduced domain shift compared to training using the unaltered original source scene point clouds $X_{i}^{S}$.

### 3.3. Network Structure

**Figure 2:**
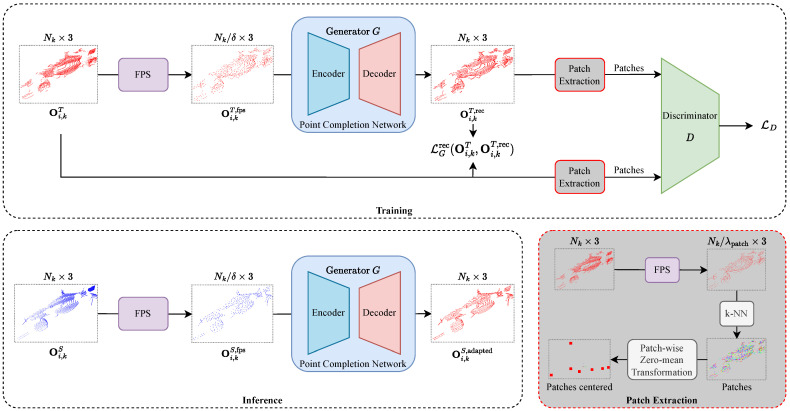
Detailed structure of our object-based point cloud domain adaptation network. The training procedure involves reducing the size of a point cloud $O_{i,k}^{T}$ by means of farthest point sampling (FPS) and then reconstructing it using the generator *G*, which is a point completion network. We employ a discriminator that uses patches with $\lambda_{\mathrm{patch}}$ points as input to further aid reconstruction. During inference, the generator *G* takes the source point clouds $O_{i,k}^{S}$ that have been downsampled using FPS as input and then adapts them locally to generate the output point clouds $O_{i,k}^{S,\mathrm{adapted}}$ in the target style.

Our domain adaptation method is based on a GAN, which includes a generator *G* and a discriminator *D*. The discriminator is only used during the training process to guide the training of the generator. The network structure and specifics for training and inference are illustrated in Figure 2. We will first discuss the input representation of the network before providing details of the generator and discriminator.

#### 3.3.1. Input Representation

Our network adapts the local structure of object point clouds, as outlined in Section 3.2. The number of points associated with each object usually decreases as the distance of the object from the LiDAR sensor increases. Consequently, our network must be able to process a varying number of input points and must be able to generate a varying number of output points. The number of points per object point cloud can be as few as one for objects that are far away, which makes it hard to adapt the domains due to the lack of local structure. Therefore, we define a minimum number of points. If an object point cloud has fewer points than this predetermined minimum, we will not use it to train our domain adaptation network.

Each input object point cloud is downsampled by a fixed downsampling factor δ using the farthest point sampling (FPS) algorithm. Depending on the choice of δ, FPS enables the preservation of the global structure of the object point cloud while removing the local structure, thus making the downsampled object point cloud domain-invariant. δ is a hyperparameter that can be tuned for each domain separately. In practice, a reasonable choice for δ is in the range of [3,10].

#### 3.3.2. Generator

The generator *G* has the task of upsampling the previously FPS downsampled object point cloud, which is either an object point cloud of the target or the source domain for training or testing, respectively. We define the upsampling factor for the generator to be the same as the downsampling factor δ, as the number of points in the output point cloud should be the same as the number of points in the input point cloud.

During training, *G* aims to reconstruct the target point cloud, thus learning to complete the local structure of the input while preserving the global structure. At inference time, the same network is used to reconstruct the FPS downsampled source object point cloud, but it reconstructs the source point cloud using the local structure of the target domain it has seen during training. Therefore, domain adaptation occurs only when making predictions during inference and not during training, as the latter is based on target domain reconstruction only.

Our method is highly scalable, as it requires a dataset of target point clouds for training, but once trained, it can adapt an unlimited number of source point clouds. Our network does not need to be trained to adapt a particular source domain, so it can adapt source data from various source domains, such as different simulations, without needing to be retrained.

We employ the state-of-the-art point completion network SeedFormer [47], which can recover regional information of local patterns in a coarse-to-fine manner. Rather than relying on the seeds generated from the global and patch features of SeedFormer, we directly use the FPS downsampled point cloud in the SeedFormer decoder. The purpose of the original SeedFormer architecture is to upsample a partial point cloud, which requires the generation of the missing elements of the point cloud. However, our FPS-downsampled point cloud already contains all the global parts of the input and output point cloud. Furthermore, the SeedFormer architecture must be modified to process a varying number of input points, necessitating alterations to the PointNet++ set abstraction (SA) layers employed in the feature extractor of SeedFormer. We modify the SA layers so that the number of query points is determined dynamically based on the number of input points instead of using a fixed number, as in the original SeedFormer architecture.

Apart from these changes, we use the original SeedFormer architecture. This implies that *G* can be substituted with any point completion or point upsampling network, as long as the requirement for a variable number of input points can be integrated.

#### 3.3.3. Patch Discriminator

In addition to the generator *G* that upsamples the input point cloud to the target style, we employ a discriminator to guide the reconstruction process and concentrate on the local aspect of reconstruction. The generator’s reconstruction attempts to balance between local and global reconstruction, which can sometimes lead to suboptimal results for certain local parts of the point clouds. This is especially noticeable in the sparse areas of the point clouds, which we will explain using the following example.

Choose a point $p_{i}$ without close neighbors in the input point cloud $O_{i,k}$. When applying FPS downsampling, this point will be chosen as a seed point due to its large distance from other points and will be included in the set of points that the generator *G* will process. During upsampling, *G* has the task of upsampling $p_{i}$ δ-times and placing δ−1 points around this seed point $p_{i}$. This patch in the input point cloud contains a single point, whereas the same patch in the output point cloud contains δ points. Since *G* attempts to reconstruct the overall structure during the training process, the patch of δ points could converge to the same coordinate as the input point $p_{i}$.

We use a discriminator *D* to tackle this issue, which assists *G* in concentrating on reconstructing local structures. *D* discriminates between point cloud patches from the input point cloud $O_{i,k}^{T}$ and the reconstructed point cloud $O_{i,k}^{T,\mathrm{rec}}$.

The extraction of the point cloud patches is an upstream process shown in Figure 2 downright. The centers of the potential patches are determined by extracting points from either $O_{i,k}^{T}$ or $O_{i,k}^{T,\mathrm{rec}}$. We use the k-Nearest Neighbor (k-NN) algorithm to identify the $\lambda_{\mathrm{patch}}$ points that are closest to each patch center. The size of each patch $\lambda_{\mathrm{patch}}$ is closely linked to the downsample and upsample factor δ, as the size of a potentially collapsing patch is usually equal to δ, as previously mentioned. The last step of patch extraction involves transforming the coordinates of each patch to have a mean of zero.

Taking the patches of $O_{i,k}^{T}$ and $O_{i,k}^{T,\mathrm{rec}}$, *D* is trained to differentiate between the original and reconstructed patches. *G* is trained adversarially and receives the negative loss of *D*.

*D* comprises two PointNet++ SA layers followed by three fully connected (FC) layers. Instead of the default ball query to sample neighboring points in the SA layers, we opted to use k-NN to sample three neighbors for each query point due to the varying density of our objects. We increase the feature dimension of each sampled point to 1024 before applying MaxPooling and feeding the 1024-dimensional global feature vector into the FC layers. We adhere to the standard practice of not including BatchNorm layers in *D* and using LeakyReLU activation functions in the FC layers. In Section 5.3, we investigate the impact of including *D* in the training phase on our results.

### 3.4. Loss Function

The total loss of our GAN architecture $L_{\mathrm{total}}$ is a linear combination of the generator loss $L_{G}$ and the discriminator loss $L_{D}$

(1) $$L_{\mathrm{total}}=L_{G}+L_{D}.$$

The generator loss $L_{G}$ consists of two parts. The first part is the reconstruction loss $L_{G}^{\mathrm{rec}}$ and the second part is the adversarial loss $L_{G}^{\mathrm{adv}}$. Following [47], we use the chamfer distance (CD) to compute the reconstruction loss $L_{G}^{\mathrm{rec}}$ of *G*. The chamfer distance measures the distance between two unordered sets of points $S_{1},S_{2}\subseteq R^{3}$. It is calculated by taking the sum of the shortest distances between each point in one set and the closest point in the other set.

(2) $$\begin{array}{cc} d_{CD}\left(S_{1},S_{2}\right)=\frac{1}{2} & \left(\frac{1}{\left|S_{1}\right|}\sum_{x\in S_{1}}\min_{y\in S_{2}}{\left|\left|x-y\right|\right|}_{2}^{2}\right.\\ \; & \left.+\frac{1}{\left|S_{2}\right|}\sum_{y\in S_{2}}\min_{x\in S_{1}}{\left|\left|y-x\right|\right|}_{2}^{2}\right) \end{array}$$

As we compare the point clouds on the object-level, $L_{G}^{\mathrm{rec}}$ is defined as

(3) $$\begin{array}{c} L_{G}^{\mathrm{rec}}\left(O_{i,k}^{T,\mathrm{rec}},O_{i,k}^{T}\right)=d_{CD}\left(O_{i,k}^{T,\mathrm{rec}},O_{i,k}^{T}\right). \end{array}$$

We also experimented using the earth movers distance (EMD) but found the reconstruction results inferior to CD. The adversarial loss of the generator $L_{G}^{\mathrm{adv}}$ is calculated as

(4) LGadvOi,kT,=−logDGOi,kT,,

where $G(O_{i,k}^{T})$ represents the reconstructed point cloud $O_{i,k}^{T,\mathrm{rec}}$. If $L_{G}^{\mathrm{adv}}\left(O_{i,k}^{T}\right)$ is close to one, it implies that the generator *G* has deceived the discriminator successfully.

The loss of the discriminator $L_{D}$ is calculated as

(5) LDOi,kT,=−logDOi,kT,−log1−DGOi,kT,,

where $D(O_{i,k}^{T})$ represents the discriminator’s output when evaluating a target point cloud, which should be close to one. $D(G\left(O_{i,k}^{T}\right))$ represents the discriminator’s output when evaluating a reconstructed point cloud $O_{i,k}^{T,\mathrm{rec}}$, which should be close to zero since *G* generates it.

Inserted into Equation (1), the total loss $L_{\mathrm{total}}$ can be calculated as

(6) Ltotal=LGrecOi,kT,rec,Oi,kT+LGadvOi,kT,+LDOi,kT,=dCDOi,kT,rec,Oi,kT−logDGOi,kT,−logDOi,kT,−log1−DGOi,kT,.

## 4. Experimental Setup

In this chapter, we provide an overview of the experimental setup by introducing our dataset, describing the model configuration, and providing details of the metrics used for the evaluation.

### 4.1. Dataset

The focus of our work is on a domain adaptation algorithm that minimizes the LiDAR sim-to-real domain shift. This requires a dataset with data from the source and target domains, whereas the source data are generated in simulation and the target data are captured in the real world. The distinguishing feature of the real-world target domain, as opposed to the simulated source domain, is predominantly its complex and variable sensor noise characteristics. Real-world environments present a challenging array of sensor noises that are typically absent or greatly simplified in simulation settings. This noise can originate from a variety of sources, including environmental factors such as rain, fog, or varying light conditions, and from inherent imperfections and aging of the sensor hardware. In contrast, simulated data often lack this level of noise variability, offering overly clean and consistent sensory inputs.

Our domain adaptation method focuses on adapting the local structure of object point clouds. Therefore, to satisfy the need for a separate examination of the global and local domain shifts, we require a dataset in which the global domain shift is minimized. A dataset that meets this requirement is the Sim-to-Real Distribution-Aligned Dataset introduced in [18]. This dataset consists of two subsets and is described briefly in the following. For a detailed description, we refer to [18]. One subset was captured in the real world and automatically annotated using the GPS trajectories of the vehicles involved. It contains scenarios captured during the Indy Autonomous Challenge in Las Vegas on a race track with AV-21 race cars. The other subset is a scenario-identical digital counterpart generated in simulation using the same GPS trajectories. This digital counterpart includes the same scenarios, objects, and environment as the real-world counterpart. The subsets are distribution-aligned, that is, an almost scenario-identical simulated counterpart exists for every real-world point cloud. Therefore, this dataset is uniquely suitable for our study, as it minimizes the global domain shift, allowing us to focus on and accurately evaluate the adaptation of local structures. Other available dataset pairs of real-world and simulated data do not provide the same level of global alignment, making them less suitable for isolating and assessing the effectiveness of adaptation of local structures. We recognize the restriction of our current approach and intend to broaden our technique to encompass a wider range of datasets in the future, thus increasing the generality and applicability of our study.

We use this dataset both for training our object-based domain adaptation method and to evaluate the performance of the adaptation by training object detection networks using the adapted scene point clouds $X_{i}^{S,\mathrm{adapted}}$.

The former requires extracting the objects as explained in Section 3.2 and shown in Figure 1. To that end, we extract all object point clouds with at least 512 points per object from both the real-world and simulated datasets. We set this minimum number of points per object since object point clouds with fewer points than this minimum number of points are sparse and barely contain any local information. After extracting the object point clouds from the scene point clouds, our object dataset contains 528, 14, and 14 object point clouds for training, validation, and testing, respectively. The validation and testing splits of this dataset are intentionally much smaller compared to the training split because the target object dataset is exclusively used for training purposes, i.e., the target object dataset is used to train and validate our domain adaptation network and to test reconstruction after training.

Once training is complete, the entire source object dataset consisting of 556 objects is used to generate domain-adapted object point clouds $O_{i,k}^{S,\mathrm{adapted}}$, which are then placed back into their original positions in the source scene point clouds $X_{i}^{S}$.

In the remainder of this work, we will refer to the domain-adapted dataset as *sim-to-real*, meaning that it has been adapted from the source (*sim*) to the target (*real*) dataset.

**Benchmark.** To further benchmark the performance of our domain adaptation method with a baseline method, we employ a noise module to the virtual sensor model in simulation. Using this noise module, we create a new simulation dataset with different LiDAR characteristics but the same scenarios as the original *sim* dataset. In particular, we add Gaussian noise with a standard deviation of 2 cm to each LiDAR ray in the longitudinal direction, similar to [18]. To make it comparable to our approach, which only adapts the objects and not the entire scene, the noise of the sensor model is only applied to all points that hit objects. This baseline dataset is referred to as *sim-noise*.

### 4.2. Model and Training Setup

We train our GAN architecture end-to-end using the PyTorch framework, with the AdamW optimizer and a learning rate of 10^−4^. The patch size $\lambda_{\mathrm{patch}}$ is equal to the downsampling factor δ, as described in Section 3.3.3. When training sim-to-real domain adaptation, the downsampling factor δ is set to seven, and when training real-to-sim domain adaptation, it is set to five. As the input number of points is variable, the batch size is limited to one. Each configuration is trained for 100 epochs.

We assess our domain adaptation using object detectors. Since the training of these networks is non-deterministic, we train each configuration five times and report the mean and standard deviation of each metric.

### 4.3. Metrics

We measure the performance of our domain adaptation network by training 3D LiDAR object detectors on the source, target, and adapted datasets. We then evaluate each trained network on the target dataset, as was performed in [17,18]. We assess both the *sim-to-real* and the *real-to-sim* directions of the domain shift. To validate our method for a point-based and a voxel-based object detection approach, we use PointRCNN [48] and PointPillars [49]. Both networks use the same configuration as in [18]. Furthermore, we use the same test split of 1000 point clouds for evaluation. However, instead of using the entire training split, we only use the point clouds that include adapted objects. We report the 3D average precision (AP) and recall as metrics. For each metric, we assess the performance at two different intersection over union (IoU) thresholds, 50% and 70%. These are referred to as 3D AP (0.5) or Recall (0.5) and 3D AP (0.7) or Recall (0.7), respectively. In addition, we report the AP for the full range, which encompasses an object distance of up to 100 m, as well as for the close range $r_{1}=\left[0.0\;m,33.3\;m\right)$, similar to [18]. Especially, $r_{1}$ is relevant since most adapted objects are within this range due to the minimum number of points needed by the domain adaptation network.

In addition to the quantitative evaluation, we further assess the performance of our domain adaptation method qualitatively by visually inspecting the adapted point clouds in comparison to the source and target domains. To this end, we visualize the 3D point clouds of selected objects of the *real*, *sim*, and *sim-to-real* datasets and also visualize a cropped part of the objects to highlight the local structure differences. Additionally, we aggregate multiple point clouds of each domain into a single point cloud, allowing a qualitative comparison of the entire object point cloud. As another qualitative metric, we visualize the t-distributed stochastic neighbor embedding (t-SNE) [23] using the high-dimensional latent features of the object detection networks, generated during inference when passing point clouds of different domains through the networks.

## 5. Results

### 5.1. Quantitative Evaluation

**Figure 3:**
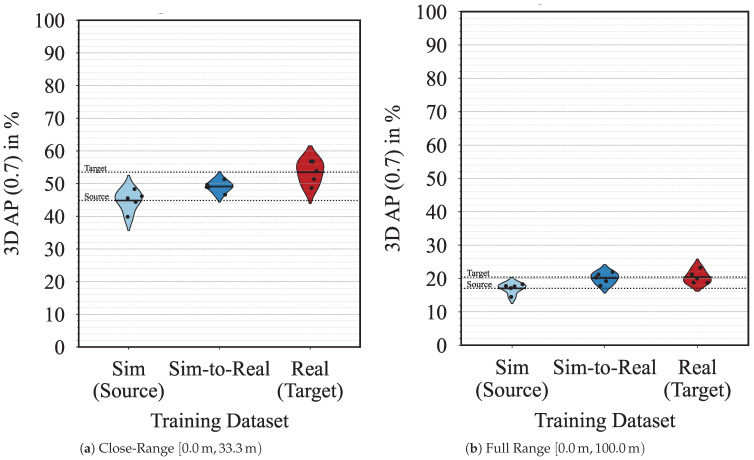
3D average precision (AP) with IoU 70% for PointRCNN trained with *sim*, *sim-to-real*, or *real* data and evaluated on *real* data (target). The horizontal lines mark the mean AP, and the five points mark the individual five training runs per train-test pairing.

The bean plots in Figure 3 show the quantitative results of the object detection network PointRCNN trained on different datasets. Each bean represents the five identical training runs of a specific dataset, whereas all training runs are evaluated on the test split of the *real* dataset. We further split the evaluation into different ranges, that is the close-range $\left[0.0\;m,33.3\;m\right)$ and the full range $\left[0.0\;m,100.0\;m\right]$. When analyzing the close-range, the 3D AP (0.7) of *real* (53.48%) is higher than the performance of *sim* (44.85%), highlighting the sim-to-real domain shift of 8.63% we aim to minimize using domain adaptation. In the same range, our domain-adapted *sim-to-real* dataset achieves a 3D AP of (49.12%), showing a considerable minimization of the sim-to-real domain shift from 8.63% to 4.36%, a reduction of almost 50%. Similar results can also be seen for the full range, PointPillars, and 3D AP (0.5) in Table 1. In Table 2, we show the recall for both IoU thresholds, corresponding with the 3D AP results.

Nevertheless, a remaining domain shift of 4.36% still exists in the *sim-to-real* adapted dataset. This can be explained by the fact that our method adapts only the object point clouds but still uses the remainder of the source scene point cloud, contributing to the domain shift. Nevertheless, we want to mention that our domain adaptation method performs better than the source dataset in all scenarios.

When comparing our sim-to-real domain adaptation method with the baseline approach introduced in the *sim-noise* dataset, our method performs better for both PointRCNN and PointPillars in terms of 3D AP and recall. Our method minimizes the sim-to-real domain shift more than the baseline approach. Only in one instance does *sim-noise* outperform our *sim-to-real* dataset, and that is for PointPillars in close-range 3D AP (0.5). The lower false positive rate of *sim-noise* compared to *sim-to-real* is likely due to its more consistent noise pattern, resulting in better performance at lower IoU thresholds.

### 5.2. Qualitative Evaluation

**Figure 4:**
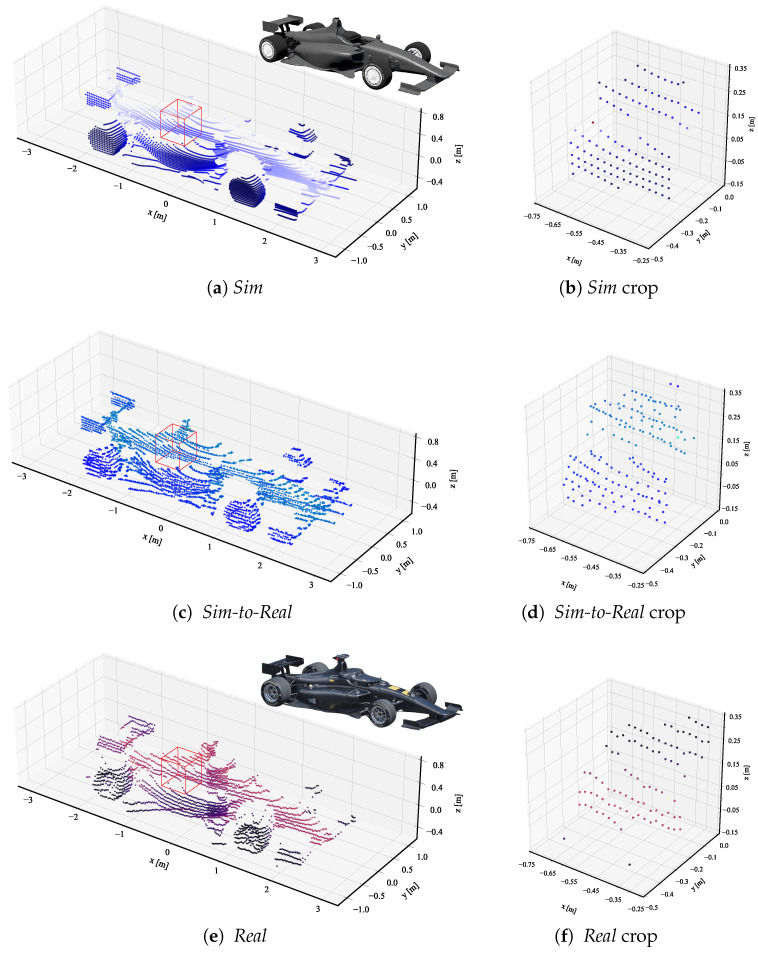
Single object point clouds of *sim*, *sim-to-real*, and *real* datasets for comparison of the domain adaption method. In (**b**,**d**,**f**) we provide a crop of the red boxes depicted in (**a**,**c**,**e**), respectively, for a detailed view of the local structure. Blue shades represent simulated point clouds and red shades represent real-world point clouds. For reference, we include the 3D model and picture of the object in (**a**,**e**), respectively.

As explained in Section 4, we further analyze the adapted point clouds qualitatively by visualizing samples of object point clouds. Figure 4 shows three selected point clouds from the *sim*, *sim-to-real*, and *real* datasets, each with a similar number of points. The point color is consistent with the rest of our paper, with blue hues representing simulated point clouds and red hues representing real-world point clouds. The color gradient within each point cloud correlates with the object’s y-axis, visually encoding the y-axis dimension through color intensity. Along the object point clouds, we visualize a cropped section of the object point clouds to analyze the local structure in detail. The object point clouds of the *sim* and *real* dataset in Figure 4a,e are clearly distinguishable, highlighted by the smooth contours of the *sim* point clouds compared to the noise silhouette of the *real* point cloud. This local difference is especially visible in the cropped sections in Figure 4b,f.

Figure 4c shows the *sim-to-real* adapted object point cloud. The cropped section in Figure 4d highlights the successful domain adaptation on a local level, introducing a noisy characteristic similar to the *real* cropped section.

**Figure 5:**
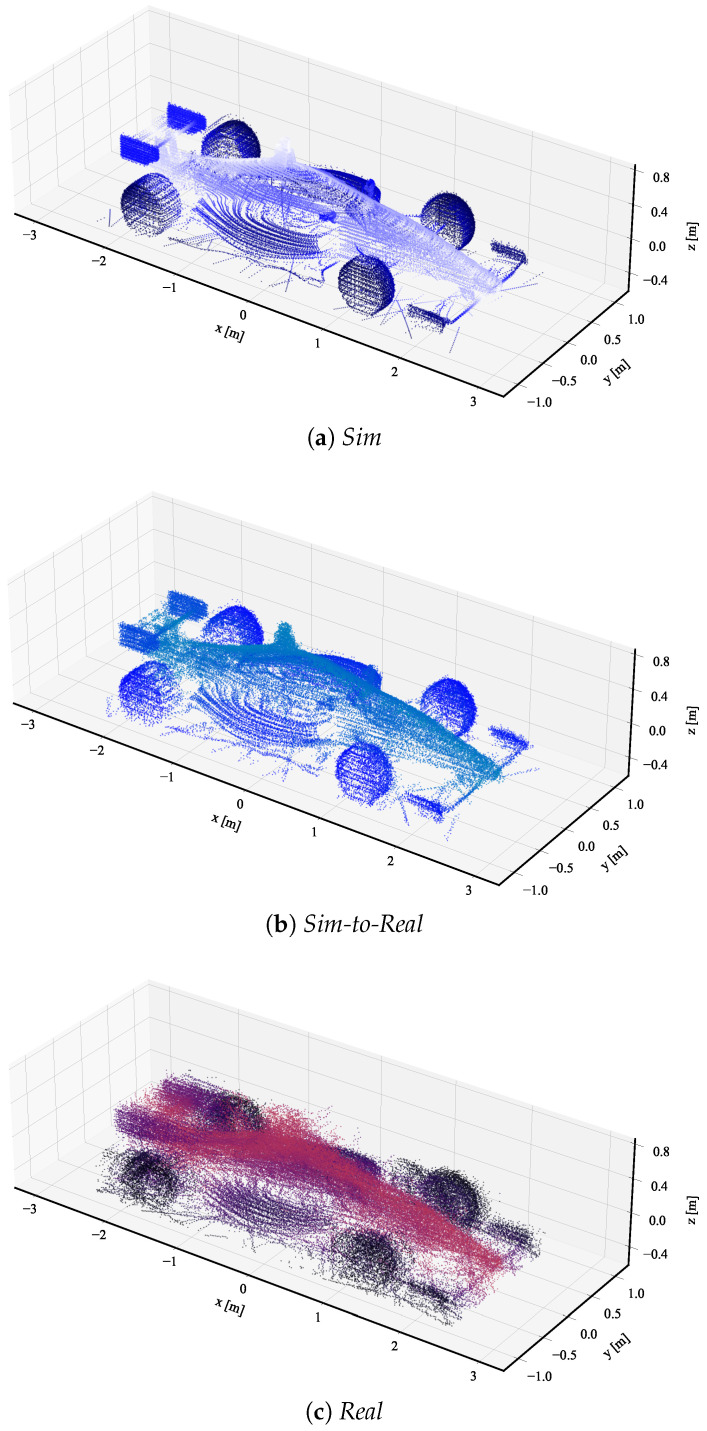
Aggregated normalized object point clouds of *sim* (blue), *sim-to-real* (blue), and *real* (red) datasets. Each aggregated object point cloud consists of 50 randomly selected individual point clouds.

The aggregated object point clouds are compared in Figure 5. In these plots, 50 randomly selected point clouds of each domain are first normalized and then aggregated to generate a single aggregated object point cloud per domain. This allows for a comparison of all parts of the objects. When comparing the *sim* and the *sim-to-real* aggregated point clouds in Figure 5a,b, respectively, the *sim-to-real* clearly shows a noisy characteristic on the local level similar to the real point cloud in Figure 5c. This further emphasizes a successful sim-to-real domain adaptation.

**Figure 6:**
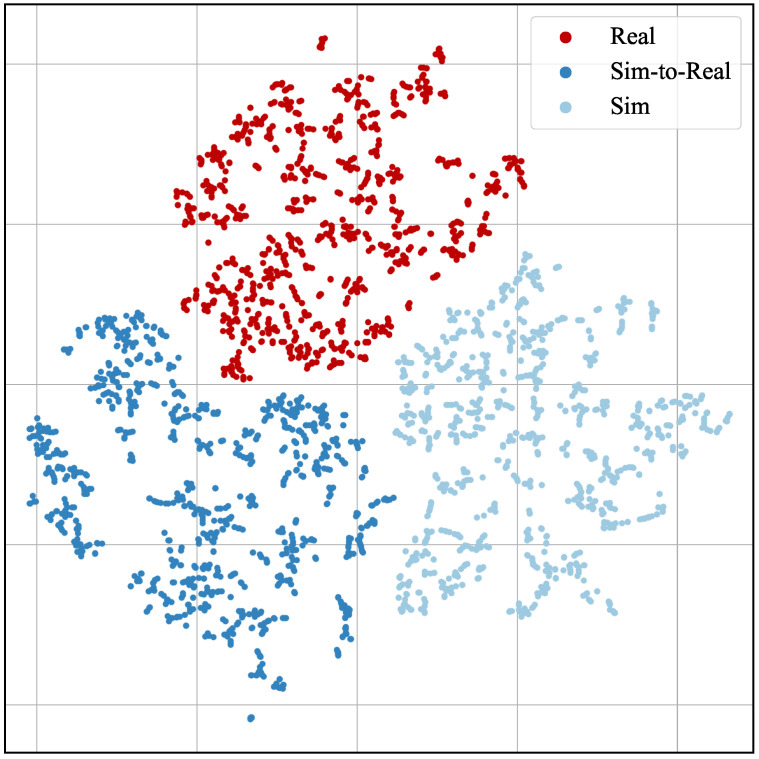
T-SNE plot of the latent feature space of PointPillars trained on *real*, *sim*, or *sim-to-real* data. Each point visualizes a feature vector generated by network inference with a single point cloud of the *real* test set.

A t-SNE plot of the latent feature space of PointPillars trained on *real*, *sim-to-real*, and *sim* is depicted in Figure 6. Each point is a 2D representation of the high-dimensional latent feature space of PointPillars when tested on the *real* dataset. For a perfect domain adaptation, the expectation would be that the cluster of the *sim-to-real* dataset merges with the cluster of the *real* dataset. Since three distinct clusters are visible for each training dataset, this shows that a domain shift between the *sim-to-real* and the *real* dataset still exists even after our domain adaptation. This is in agreement with the quantitative results. However, t-SNE cannot quantify the magnitude of the remaining domain shift.

### 5.3. Ablation Study

To demonstrate the sensitivity of certain parameters of our domain adaptation method, we conduct an ablation study. In detail, we analyze the impact of the downsampling factor δ and the impact of adversarial training using the discriminator.

**Figure 7:**
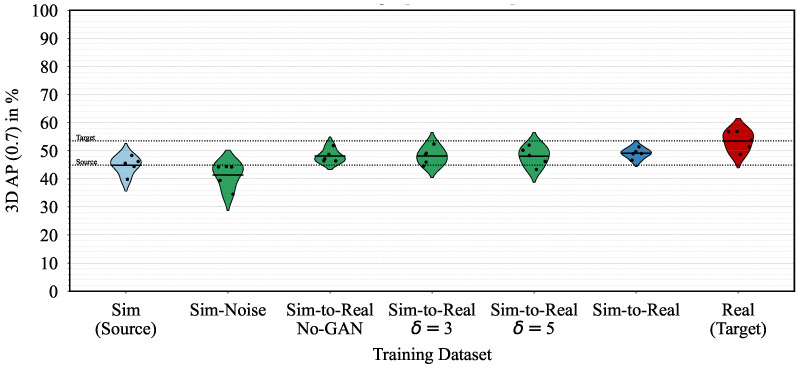
3D average precision (AP) with IoU 70% for PointRCNN trained with *sim*, *real*, multiple *sim-to-real* variants, or *sim-noise* data and evaluated on *real* data (target) in close-range $\left[0.0\;m,33.3\;m\right)$. For the *sim-to-real* variants, we alter the downsampling factor δ from its default value seven to five or three, respectively. Furthermore, we analyze the performance of our domain adaptation method without adversarial training, i.e., without the discriminator (*Sim-to-Real No-GAN*). The horizontal lines mark the mean AP, and the five points mark the individual five training runs per train-test pairing.

In the first experiment, we alter the downsampling factor δ from the original *sim-to-real* setting of δ=7 to five and three. After training our domain adaptation network with these settings, we create two adapted datasets *sim-to-real δ=5* and *sim-to-real δ=3*. These datasets are also evaluated using the two object detection networks PointRCNN and PointPillars. Figure 7 presents the 3D AP (0.7) results of these experiments for PointRCNN and close range, while the remaining results can be found in Table 1 and Table 2. When choosing a lower downsampling factor of either δ=5 or δ=3, the mean 3D AP (0.7) decreases by more than one percent to 48.00% or 48.10%, respectively, in comparison to δ=7 with 49.12%. This observation is particularly noticeable when examining PointPillars. Recall also demonstrates a decrease in performance as the downsampling factor decreases. It is reasonable to assume that a larger downsampling factor will give the generator more freedom to generate meaningful adapted point clouds. However, a larger downsampling factor also decreases the number of points in the domain-invariant input point cloud, which can cause the adaptation to be more focused on the global space than the local level.

We conduct a second experiment to analyze the performance of our network trained without the adversarial setting. Therefore, we remove the discriminator from the architecture and train using the generator reconstruction loss $L_{G}^{\mathrm{rec}}$ only. This setting allows us to quantify the influence of the discriminator. We refer to the resulting sim-to-real adapted dataset as *sim-to-real no-GAN*. As shown in Figure 7, the mean 3D AP (0.7) of *sim-to-real no-GAN* (48.09%) is greater than the mean 3D AP of *sim* (44.85%), thus minimizing the sim-to-real domain shift. However, in comparison to the default *sim-to-real* (49.12%) with adversarial training, *sim-to-real no-GAN* does not perform as well, thus demonstrating the advantage of using a patch discriminator with adversarial training. It is worth noting that in certain settings, such as PointRCNN’s 3D AP (0.5) or Recall (0.5), the *sim-to-real no-GAN* approach outperforms the default *sim-to-real* method. Despite this, most metrics demonstrate improved results when adversarial training is used.

Since our method is applicable for any combination of source and target datasets, we also analyze the performance of *real-to-sim*, i.e., adapting *real* point clouds to *sim* point clouds. The results of this analysis are summarized in Table 3. The adapted *real-to-sim* dataset consistently outperforms the source dataset, showing a reduced real-to-sim domain shift, albeit the magnitude of reduction differs between PointRCNN and PointPillars.

## 6. Discussion

In the following, we discuss the limitations of our presented domain adaptation network.

First, in our method, the need for an annotated target dataset initially presents itself as a limitation, as it requires access to labeled target domain data for training. However, this approach has been strategically chosen to enhance the model’s scalability and domain independence. Once trained with the target domain data, our network is capable of adapting an unlimited number of source domain point clouds without the need for retraining for each new domain. This means that, while the initial requirement for labeled target data may seem restrictive, it actually opens up a pathway for extensive and versatile application of the model across various source domains. The use of a limited set of labeled target data facilitates the adaptation of a much larger set of source data, thus maximizing the utility and reach of our method in diverse real-world scenarios.

Second, instead of adapting entire scene point clouds, we only adapt the relevant objects, i.e., the objects detected by the perception task. This is not just a restriction, but rather a deliberate decision. Focusing on object-level adaptation aligns with the practical and computational limitations of processing large scene point clouds. Scene point clouds are typically too large for efficient GPU processing and are more sparse compared to object point clouds, which can impact the effectiveness of domain adaptation. Moreover, our generator *G*, originally designed for point cloud completion, is better suited for processing single objects rather than entire scenes. Adapting entire scenes with our method could risk losing important global structures in the objects due to the constraints of downsampling.

This requires an annotated target dataset, as we need target object point clouds to train our domain adaptation network. Once our domain adaptation network is trained, we do not need target labels, enhancing the method’s scalability and enabling it to adapt an unlimited number of annotated source point clouds from any source domain without retraining for each source domain, as explained in the first limitation. Although focusing on object-level adaptation means that we cannot fully eliminate the domain shift, it allows us to maximize the precision and effectiveness of our adaptation within the most critical aspects of the data, which is reflected in the results.

Third, our method can handle a variable number of points in the object point cloud but still requires a minimum number of points, as sparse object point clouds do not contain meaningful information on local structures.

In future work, the current limitations can be resolved by extending our method to adapt entire scene point clouds. In addition, our method can be transferred to public real-world and simulated datasets, such as KITTI and CARLA. These datasets are not distribution-aligned. Hence, they contain a global domain shift in addition to the local domain shift due to differences in objects, scenarios, environment, etc. Therefore, our method cannot fully eliminate the domain shift in these datasets. However, the use of such diverse datasets will allow us to further evaluate and enhance the generalizability of our domain adaptation method.

Although our current study relies on the Sim-to-Real Distribution-Aligned Dataset, specifically chosen for its minimal global domain shift, we recognize the importance of testing our method across a variety of datasets to establish broader applicability and robustness. By expanding our research to include datasets that present both global and local domain shifts, we aim not only to validate our method in more complex scenarios but also to refine it to address a wider range of domain adaptation challenges. Since our method can reduce the domain shift on our distribution-aligned dataset, we expect to also reduce the total domain shift on these public datasets by focusing on the reduction in the local domain shift. Another area of research is the intensity channel of the LiDAR point clouds, which we currently neglect but want to include in future work.

## 7. Conclusions

In this paper, we have introduced a novel domain adaptation network for 3D LiDAR object detection, achieving significant advancements in minimizing the sim-to-real domain shift. Our adversarial-based network architecture, incorporating a point cloud completion network for generation and a PointNet++-inspired discriminator, has demonstrated a notable reduction in the domain shift, quantified by a decrease in the domain shift from 8.63% to 4.36% 3D AP.

Key findings of our research include the ability of the network to adapt object point clouds with high fidelity to the target domain’s local structure. This achievement is further underscored by our network’s outperformance of baseline models. Additionally, we have established the importance of the downsampling factor and the adversarial training strategy in improving domain adaptation.

Although our approach necessitates an initial annotated target dataset and focuses on object-level adaptation, these aspects are balanced by the method’s scalability and precision. The limitations noted pave the way for future work, where we aim to extend our methodology to entire scene point clouds and explore its applicability on diverse datasets, like KITTI and CARLA, as well as incorporating the intensity channel of LiDAR data.

In conclusion, our domain adaptation network marks a significant step forward in the field of robust 3D object detection using domain adaptation.

## Figures and Tables

**Table 1:** Sim-to-Real: 3D average precision for IoU thresholds 70% and 50%, denoted as 3D AP (0.7) or 3D AP (0.5), respectively. The networks PointRCNN and PointPillars were trained five times using the indicated train dataset and evaluated on the test split of the *real* dataset. ↑: Higher values are better, bold values mark the best value of each category.

Network	Train Dataset	3D AP (0.7) ↑		3D AP (0.5) ↑	
		**Close-Range**	**Full Range**	**Close-Range**	**Full Range**
**PointRCNN**	**Sim (Source)**	44.85 ± 2.82	17.06 ± 1.34	52.48 ± 1.42	24.11 ± 2.31
	Sim-Noise	41.34 ± 3.89	15.58 ± 2.83	49.14 ± 5.07	22.17 ± 4.47
	Sim-to-Real No-GAN	48.09 ± 2.05	20.03 ± 3.29	**57.94 ± 0.81**	**30.58 ± 1.75**
	Sim-to-Real δ=3	48.10 ± 2.72	**20.39 ± 3.44**	55.44 ± 1.68	28.67 ± 3.93
	Sim-to-Real δ=5	48.00 ± 3.03	20.19 ± 2.65	56.36 ± 1.14	28.89 ± 2.43
	Sim-to-Real	**49.12 ± 1.53**	20.10 ± 1.46	56.51 ± 1.31	29.82 ± 1.99
	**Real (Target)**	53.48 ± 3.15	20.40 ± 1.71	59.17 ± 1.88	28.46 ± 4.09
**PointPillars**	**Sim (Source)**	26.39 ± 0.00	9.85 ± 0.00	63.51 ± 0.00	23.75 ± 0.00
	Sim-Noise	31.46 ± 0.00	11.80 ± 0.00	**67.16 ± 0.00**	28.45 ± 0.00
	Sim-to-Real No-GAN	26.33 ± 0.00	10.90 ± 0.00	63.77 ± 0.00	29.20 ± 0.00
	Sim-to-Real δ=3	28.03 ± 0.00	10.86 ± 0.00	61.47 ± 0.00	**30.01 ± 0.00**
	Sim-to-Real δ=5	30.69 ± 0.00	11.91 ± 0.00	61.10 ± 0.00	28.79 ± 0.00
	Sim-to-Real	**38.03 ± 0.00**	**14.41 ± 0.00**	64.11 ± 0.00	29.31 ± 0.00
	**Real (Target)**	51.32 ± 0.00	18.33 ± 0.00	81.52 ± 0.00	32.62 ± 0.00

**Table 2:** Sim-to-Real: Recall for IoU thresholds 70% and 50%, denoted as Recall (0.7) or Recall (0.5), respectively. The networks PointRCNN and PointPillars were trained five times using the indicated train dataset and evaluated on the test split of the *real* dataset. ↑: Higher values are better, bold values mark the best value of each category.

Network	Train Dataset	Recall (0.7) ↑	Recall (0.5) ↑
**PointRCNN**	**Sim (Source)**	32.22 ± 2.11	44.52 ± 2.91
	Sim-Noise	29.92 ± 1.20	43.04 ± 1.44
	Sim-to-Real No-GAN	32.18 ± 1.88	**44.20 ± 1.51**
	Sim-to-Real δ=3	31.32 ± 3.56	42.76 ± 3.05
	Sim-to-Real δ=5	32.12 ± 1.64	42.66 ± 0.78
	Sim-to-Real	**33.00 ± 0.73**	43.22 ± 0.88
	**Real (Target)**	38.50 ± 2.32	64.32 ± 1.63
**PointPillars**	**Sim (Source)**	15.50 ± 0.00	26.40 ± 0.00
	Sim-Noise	18.00 ± 0.00	32.90 ± 0.00
	Sim-to-Real No-GAN	18.70 ± 0.00	34.80 ± 0.00
	Sim-to-Real δ=3	15.90 ± 0.00	33.10 ± 0.00
	Sim-to-Real δ=5	19.40 ± 0.00	36.20 ± 0.00
	Sim-to-Real	**21.90 ± 0.00**	**36.40 ± 0.00**
	**Real (Target)**	22.10 ± 0.00	34.40 ± 0.00

**Table 3:** Real-to-Sim: 3D average precision for IoU thresholds 70% and 50%, denoted as 3D AP (0.7) or 3D AP (0.5), respectively. The networks PointRCNN and PointPillars were trained five times using the indicated train dataset and evaluated on the test split of the *sim* dataset. ↑: Higher values are better.

Network	Train Dataset	3D AP (0.7) ↑		3D AP (0.5) ↑	
		**Close-Range**	**Full Range**	**Close-Range**	**Full Range**
**PointRCNN**	**Real (Source)**	55.73 ± 4.78	22.69 ± 3.16	62.66 ± 2.38	32.87 ± 4.80
	Real-to-Sim	55.94 ± 3.68	22.72 ± 3.42	68.43 ± 4.07	40.04 ± 7.93
	**Sim (Target)**	65.37 ± 2.69	26.98 ± 1.89	71.99 ± 2.23	41.19 ± 1.56
**PointPillars**	**Real (Source)**	23.46 ± 0.00	8.27 ± 0.00	77.04 ± 0.00	28.56 ± 0.00
	Real-to-Sim	32.03 ± 0.00	13.08 ± 0.00	85.79 ± 0.00	34.57 ± 0.00
	**Sim (Target)**	95.94 ± 0.00	33.93 ± 0.00	99.89 ± 0.00	39.26 ± 0.00

## Data Availability

The data presented in this study are openly available in https://doi.org/10.14459/2023mp1695833 (accessed on 21 November 2023).

## Abbreviations

The following abbreviations are used in this manuscript:

AdaBN	Adaptive Batch Normalization
AP	Average Precision
AV	Autonomous Vehicle
CD	Chamfer Distance
EMD	Earth Movers Distance
FPS	Farthest Point Sampling
GAN	Generative Adversarial Network
IoU	Intersection over Union
LiDAR	Light Detection and Ranging
SA	Set Abstraction
t-SNE	t-distributed Stochastic Neighbor Embedding
VAE	Variational Autoencoder
